# The STarT back tool in chiropractic practice: a narrative review

**DOI:** 10.1186/s12998-017-0142-2

**Published:** 2017-04-21

**Authors:** Yasmeen Khan

**Affiliations:** Palmer College of Chiropractic Center for Chiropractic Research, 741 Brady Street, Davenport, IA 52803 USA

**Keywords:** STarT back tool, Chiropractic, Psychosocial, Stratified care, Prediction, Care setting

## Abstract

**Background:**

The Keele STarT Back Tool was designed for primary care medical physicians in the UK to determine the risk for persistent disabling pain in patients with musculoskeletal pain and to tailor treatments accordingly. In medical and physical therapy settings, STarT Back Tool’s tailored care plans improved patients’ low back pain outcomes and lowered costs.

**Objective:**

Review studies using the STarT Back Tool in chiropractic patient populations.

**Methods:**

PubMed, The Cochrane Library, Index to Chiropractic Literature, and Science Direct databases were searched. Articles written in English, published in peer-reviewed journals, that studied the STarT Back Tool in patients seeking chiropractic care were included.

**Results:**

Seven articles were selected based on inclusion and exclusion criteria. The STarT Back Tool was feasibly incorporated into 19 chiropractic clinics in Denmark. Total STarT Back 5-item score correlated moderately with total Bournemouth Questionnaire score. Two studies reported that the STarT Back Tool’s predictive ability was poor, while another reported that the tool predicted outcomes in patients scoring in the medium and high risk categories who completed the STarT Back 2 days after their initial visit. A study examining Danish chiropractic, medical and physical therapy settings revealed that only baseline episode duration affected STarT Back’s prognostic ability across all care settings. The tool predicted pain and disability in chiropractic patients whose episode duration was at least 2 weeks, but not in patients with an episode duration <2 weeks.

**Conclusion:**

While the STarT Back Tool can be incorporated into chiropractic settings and correlates with some elements of the Bournemouth Questionnaire, its prognostic ability is sometimes limited by the shorter low back pain episodes with which chiropractic patients often present. It may be a better predictor in patients whose episode duration is at least 2 weeks. Studies examining outcomes of stratified care in chiropractic patients are needed.

## Background

In the past 2 decades, health professionals have become increasingly aware of the biopsychosocial nature of sickness and disease, and more specifically, the biopsychosocial nature of pain [1–4]. Screening for specific biopsychosocial predictors of long-term disability is valuable to both clinicians and patients [5].

A tool known for its brevity, ease of use, and ability to detect multiple predictors of persistent disabling back pain is the Keele STarT Back Screening tool (SBT) [6, 7]. It was initially created to inform clinical care pathways and referral routes for patients with low back pain (LBP) seeking care from primary medical physicians [7, 8]. It was developed and validated within a cohort of patients with acute, subacute or chronic pain, with or without referred pain to the lower extremity [8]. The SBT contains 9 questions that detect musculoskeletal pain symptoms, function, fear avoidance behavior, catastrophization, anxiety, and depression [7]. The SBT appeals to clinicians and patients because it takes 2–5 min to complete, yet captures information about potentially modifiable risk factors [9]. The SBT is valid and reliable in detecting multiple factors that place acute back pain patients at risk for persistent disabling pain [4, 9].

The SBT score categorizes patients into 3 risk levels for persistent symptoms: low, medium, and high [7, 8]. The goal of this tool—and its accompanying tailored treatment strategies—is to identify patients at risk for persistent symptoms and to define management protocols tailored to each risk subgroup. Matching patient management to subgroup classification is referred to as a “stratified care” approach [5].

The STarT Back protocol for patients with low risk entails educating the patient about pain, encouraging movement and functional activities, reassuring the patient that the prognosis is good, and not providing any further treatment at that time. Patients with medium risk are referred for a course of physiotherapy that might include manual therapy, specific exercises, or general functional activities to prevent disability. Patients with high risk are referred to a physiotherapist for a combination of manual therapy or exercises, and a cognitive behavioral approach to overcoming psychosocial barriers to recovery [5, 7, 9–13].

Participants in primary care medical and physical therapy settings who received care according to STarT Back stratified care plans incurred lower medical costs and better clinical outcomes than those receiving usual care for nonspecific low back pain [5, 12, 13]. The savings of using stratified care in UK physical therapy settings was approximately £34 per patient (21 US dollars in 2009), and the savings to society was £675 per patient (425 US dollars in 2009) [5].

Thus far, evidence is inconclusive for the benefit of using formal instruments or less formal clinical prediction rules to predict who will benefit from spinal manipulative therapy [14–16]. It’s also unclear whether the SBT would be useful in predicting future outcomes or if stratified care approaches would improve outcomes in patients seeking care from a doctor of chiropractic. Therefore, the purpose of this narrative review was to examine literature on the use of the SBT in chiropractic settings to gain insight about its ability to predict future pain and disability, as well as its ability to inform treatment plans in people presenting for chiropractic care.

## Methods

### Search strategy

A search of PubMed, The Cochrane Library, Index to Chiropractic Literature (ICL), and Science Direct databases was conducted without limits through March 29^th^, 2016. Preliminary searching of the terms *start back tool* on PubMed was done to gauge the number of studies conducted on the SBT across disciplines. This preliminary search yielded 78 articles. The search terms used in PubMed, The Cochrane Library and Index to Chiropractic Literature were *start back screening tool* AND *chiropractic*. This same strategy executed in Science Direct yielded 323 papers, with a more targeted search (“*start back screening tool*” AND *chiropractic*) narrowing the yield to 11 articles. On April 2, 2017, the following additional search terms were used in PubMed to ensure rigor: (*“start back screening tool”* AND *chiropractic*; yield = 4, no new articles meeting inclusion criteria), (*start back tool* AND *chiropractic*; yield = 11; no new articles), (“*start back tool*” AND *chiropractic*; yield = 8, no new articles meeting inclusion criteria), and (“*start back tool*” AND chiropract*; yield = 8, no new articles meeting inclusion criteria).

### Article selection

**Fig. 1:**
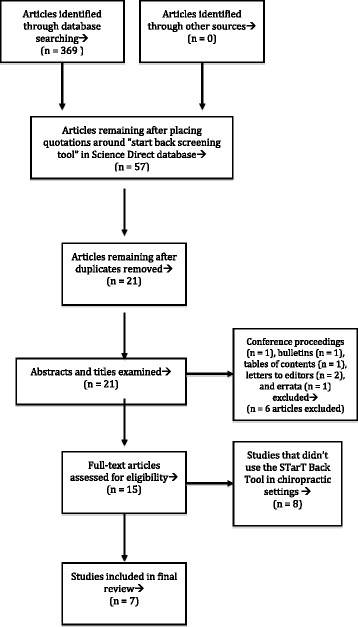
Article exclusion process

After the 4 databases were searched, yields were examined. Figure 1 summarizes the article selection process. Articles were first screened by reading the abstracts to ensure studies were conducted in chiropractic settings and that the terms *STarT Back Tool* or *STarT Back Screening Tool* were mentioned as a part of the study design. If those terms weren’t found in the abstract, the full manuscripts were retrieved, and the methods sections were analyzed for the same SBT terms. Articles that met the inclusion criteria were analyzed. Inclusion and exclusion criteria are described in Table 1.

**Table 1:** Summary of inclusion and exclusion criteria

Inclusion criteria	Exclusion criteria
Articles were published in English	Articles not published or translated into English
Articles from peer reviewed journals	Conference proceedings
All methodological designs were accepted	Letters to the editor or erratum
Studied were done in chiropractic settings	
STarT Back Tool was used in the study design	

### Data synthesis

This narrative review did not systematically synthesize the data, nor appraise the quality of the included studies prior to inclusion. Rather, the titles, authors, respective years of publication, objectives, study designs, number of participants, settings and locations, sample characteristics, primary outcomes, main results, and limitations were extracted.

## Results

### Search results

PubMed (5 searches yielding 41 articles), Cochrane Library (1 article), Index to Chiropractic Literature (4 articles), and the modified Science Direct (11 articles) searches yielded 57 articles. Seven articles were chosen for review after removing duplicate articles (*n* = 36) and those that didn’t fit inclusion criteria (*n* = 14). Table 1 details inclusion and exclusion criteria and Fig. 1 summarizes the article exclusion process.

### Main outcomes

Table 2 describes the main outcomes of each study in order of publication year. Four of the 7 studies examined aspects of the SBT’s construct validity (Kongsted et al. [17], Field and Newell [18], Irgens et al. [19], and Newell et al. [20]). Four of the studies evaluated the SBT’s prognostic capacity (Field and Newell [18], Newell et al. [20], Kongsted et al. [9], and Morso et al. [21]). Two used a cross sectional study design (Kongsted et al.[17], Irgens et al. [19]), 4 used a prospective cohort design (Field and Newell [18], Newell et al. [20], Field and Newell [22], and Kongsted et al. [9]) and 1 was a secondary analysis (Morso et al. [21]).

**Table 2:** Summary of results (*n* = 5)

Publication	Objectives related to SBT	Design	Participants (n) and Setting	% of sample in each SBT risk category (if provided)	Sample Characteristics	Main Results	Limitations
Kongsted et al. 2011 [17]. Feasibility of the STarT back screening tool in chiropractic clinics: a corss-sectional study of patients with low back pain	-Test Danish patients’ ability to fill out the Danish version of the SBT ^a^ -to see if the SBT was able to identify 3 SBT subgroups in the study population -to examine differences between the SBT 3 subgroups (in age, gender, symptoms, depression, fear avoidance beliefs and catastrophic coping strategies)	Cross-sectional	*n* = 475 19 chiropractic clinics in Denmark	Low: 59% Medium: 29% High: 11%	The high risk group had the greatest number of days with LBP ^e^ in previous year and previous 2 weeks as well as the longest current episode duration. -Study population had a higher percentage of low risk participants than the population studied in SBT validation study (8) (59%) versus 47% in medical care setting).	-Primary outcomes: SBT, MDI^b^, FABQ^c^, CSQ^d^ -Positive dose-response relationship between SBT and MDI scores (5% of low risk SBT group had signs of depression compared to 31% in high risk group). -FABQ positively correlated with SBT risk groups (1% in low risk group, 31% in high risk group). -CSQ positively correlated to SBT risk group (7% in low risk group, 55% in high risk group).	Only a small percentage of participants had high scores for primary outcomes percentages and pain and disability weren’t measured.
Field and Newell 2012 [18]. Relationship between STarT Back Screening Tool and prognosis for low back pain patients receiving spinal manipulative therapy	Compare outcomes for participants in the low, medium, and high risk SBT group after a course of usual chiropractic care.	Prospective cohort	*n* = 404 6 chiropractic clinics in England	Low: 42% Medium: 32% High: 27%	Over half (56.2%) of the sample had pain for <1 month at the onset of care.	-Primary outcome: PGIC^g^ -Secondary outcomes: BQ pain subcategory and total BQ score -BQ pain and total score was strongly associated with SBT high risk group at baseline, but by the 30 day follow-up, there was no difference between SBT groups for these 2 outcomes. -SBT groupings were not statistically significantly associated with PGIC scores at any follow-up point. -When stratified by gender, males in the SBT high risk group had 3 times the odds of poor outcome compared to low risk males at 90 days. -SBT high risk group not statistically significantly associated with low pain improvement (defined as </=2 2 points on BQ pain scale). The high risk group improved just as much as the other risk groups at each follow up. -Duration of current pain episode and reoccurrence of the pain/problem for >30 days in the last year provided some prognostic ability, but variance and predictive accuracy was low.	-Patients received usual care; it is unknown whether the chiropractors were tailoring treatment for high risk patients, according to their clinical expertise. −36.5% of the sample was lost to follow-up by the 90 day endpoint.
Irgens et al. 2013 [19]. The psychometric profile of chiropractic patients in Norway and England: using and comparing the generic versions of the STarT Back 5-item screening tool and the Bournemouth Questionnaire	Examine the correlation between the SBT and BQ ^f^scores for low back pain patients presenting for chiropractic care in Norway and England.	Cross-sectional	*n* = 214 18 chiropractic clinics in Norway *n* = 186 12 chiropractic clinics in England		Episode duration for both UK and Norwegian populations: <3 weeks: 45% >12 weeks: 37% Norwegian patients were younger, less distressed by their condition, and had lower catastrophization and depression rates, but higher anxiety rates than their English counterparts.	-Positive association between BQ total score and SBT score for all areas of musculoskeletal complaint. -Each BQ question was positively associated with overall SBT score. -Strong association between the SBT depression sub-score and BQ low mood sub-score for neck pain, moderate for back pain, and low for extremity pain. -Strong association between the BQ pain sub-score and the SBT bothersomeness sub-score for back pain in Norwegian participants (this association was moderate for UK participants). -Moderate association between the BQ pain and SBT bothersomeness sub-score for neck pain in both countries. -The association between BQ and SBT anxiety sub-scores was low to moderate.	-A validated Norwegian version of the SBT was not available at the time of the study.
Newell et al. 2015 [20]. Using the STarT Back Tool: does timing of stratification matter?	Determine if categorizing participants to SBT group at care onset led to differences in prognostic accuracy compared to those categorized 2 days after their first chiropractic visit.	Prospective cohort	*n* = 749 11 chiropractic clinics in the UK	Low: 39% Medium: 37% High: 24%	Patients over 16 years of age with nonspecific LBP who completed a BQ ^f^ at the onset of care. Duration of Pain at onset: <1 mo: 43% 1–3 mo: 10% >3 mo: 47% Those in high risk SBT category were older with a more acute presentation and higher condition severity.	-Primary Outcomes: PGIC^g^ and BQ^f^ -No difference in SBT prognostic ability in participants categorized before versus after initial visit. -Medium and high risk groups had a greater change in pain and total BQ^f^ score than low risk group. -Improvement at 14 and 30 day follow-up predicted improvement at 90 day follow-up. - > 1/3 of participants changed SBT category in the time just before initial visit and 2 days post initial visit. -SBT category post initial visit was predictive of 30 day follow-up outcomes in medium risk group, when adjusting for baseline variables. -medium risk group improved more than high risk group in spite of fewer treatment visits	−58% drop out rate by 90 day follow-up -Patients were self referring for chiropractic care; results may not be generalizable to entire nonspecific LBP population.
Kongsted et al. 2016 [9]. Prediction of outcome in patients with low back pain—A prospective cohort study comparing clinicians’ prediction with those of the Start Back Tool	Determine how clinicians’ expectations performed compared to that of the SBT and to what extent combining clinicians’ expectations with the SBT increased the amount of variation explained in outcome.	Cross-sectional (secondary analysis of a 2014 study by Eirikstoft and Kongsted) and a Longitudinal component.	*n* = 859 (710 responded at 2-weeks, 676 at 3 months, 636 at 12 months) 17 chiropractic offices in Denmark		Overall group mean scores not provided in this analysis	-Primary outcomes: NRS^h^, RMDQ ^i^ -The ability of both clinicians and SBT to predict future outcomes was low. -Clinicians’ expectations combined with the SBT were slightly better at predicting activity limitation outcomes (RMDQ) than either of the two by themselves, but the proportions of those accurately predicted was still low.	-The measure of clinicians’ expectations was not validated and it is uncertain how clinicians’ define terms like “short/uncomplicated” versus “prolonged” and “long-lasting.”
Field and Newell 2016 [22]. Clinical outcomes in a large cohort of musculoskeletal patients undergoing chiropractic care in the United Kingdom: a comparison of self- and national health service-referred routes	Compare outcomes of self-referred and National Health Service-referred patients presenting for chiropractic care.	Prospective cohort	*n* = 8,222 Chiropractic clinics in the south of the UK.	National Health Service funded patients: Low: 26% Medium: 35% High: 39% Privately funded patients: Low: 46% Medium: 32% High: 22%	-National Health Service funded patients had a statistically significantly lower percentage of patients in the low risk group compared to the self-referred group. -National Health Service referred patients were more chronic, in more distress, displayed more co-morbidity, received more treatment visits, but were less likely to continue care past 30 days than self-referred patients.	-Primary Outcomes: BQ^f^and PGIC ^g^ -Group * Time interactions existed for medium and high risk SBT categories.	-Possible between-group differences in patient expectations. -Results may not be generalizable to all patients presenting for spinal care, or patients outside of the south of the UK.
Morso et al. 2016 [21]. The prognostic ability of the StarT Back Tool was affected by episode duration	To determine whether the SBT’s prognostic ability was affected by care setting, baseline episode duration, and outcome time point being predicted.	Secondary analysis of prospective cohort data	*n* = 416 17 chiropractic clinics in Denmark *n* = 265 (General medical practice) Taken from a Danish audit project *n* = 974 (secondary care outpatient medical setting) Spine Centre in Denmark *n* = 200 (physiotherapy) 27 physiotherapy offices in Denmark	Low: 52% Medium: 39% High: 10%	-There were statistically significant differences across care settings for most baseline characteristics (age, gender, current episode duration, previous LBP episodes, pain intensity, activity limitation, and SBT score). -Most chiropractic patients were younger, male, presenting with very short episode duration (across all SBT risk groups), and most likely to score in low risk SBT group. In contrast, those at the Spine Centre had the longest episode duration.	-Primary outcome: RMDQ -The prognostic ability of the high risk versus low risk SBT group was stronger for those whose episode duration was 2 weeks or longer. The prognostic ability of the medium risk group versus the low risk group was different for people whose episode duration was greater than 12 weeks. -low risk SBT groups had the lowest activity limitation scores. -Across all care settings, the SBT’s prognostic ability is weakest for those whose episode duration is < 2 weeks. -Chiropractic settings had the greatest percentage of patients with this short episode duration.	-Secondary data analysis; there may have been other variables that had an influence on outcomes. -Measurements weren’t made on all cohorts at all time points, which may have weakened the capacity to separate the effects of setting and outcome time points.

^a^SBT = STarT Back Tool

^b^MDI = Major Depression Inventory

^c^FABQ = Fear Avoidance Beliefs Questionnaire

^d^CSQ = Coping Strategies Questionnaire

^e^LBP = Low back pain

^f^BQ = Bournemouth Questionnaire

^g^PGIC = Patient Global Impression of Change

^h^NRS = Numerical Rating Scale

^i^RMDQ = Roland Morris Disability Questionnaire

The Kongsted et al. [17] study demonstrated that the SBT was feasibly incorporated into Danish chiropractic clinics and identified patients in each of the 3 risk categories. The SBT score correlated with the Major Depression Inventory, Fear-Avoidance Belief Questionnaire, and Coping Strategies Questionnaire scores, indicating that the SBT risk groups correlated with participants’ psychological distress.

The Irgens et al. [19] study demonstrated that overall SBT score correlated moderately with overall Bournemouth Questionnaire (BQ) score and higher BQ scores were associated with being distressed by the condition, indicated by a SBT psychological subscore >4. This study also described various correlations between elements of the SBT and BQ (Table 2).

In the Field and Newell [18] study, baseline SBT score correlated positively with baseline pain score (Numerical Rating Scale and Bournemouth Questionnaire); the low risk group had the lowest pain and disability at baseline while the high-risk group had the highest. However, the outcome differences between the 3 risk groups receiving chiropractic care disappeared by 30 days follow up. By the 30-day follow-up, the high risk group had improved so substantially that there was no longer a statistically significant difference between their outcomes and those in the low and medium risk groups.

The Newell et al. [20] study reported that the SBT was prognostic of future outcomes in medium and high risk patients whose scores were collected 2 days after the initial chiropractic treatment visit, suggesting that the timing of stratification plays a role in the SBTs prognostic ability. Patients in the medium risk groups showed greater improvement than the low and high risk groups at the 14 and 30 day follow ups, but not at the 90 day follow up. One-third of the sample changed SBT risk categories in the 2 days between the initial chiropractic visit and 2 days after the initial visit.

The Field and Newell [22] study compared patients who were referred by the National Health Service (NHS) in the UK to those who were self-referring for chiropractic care. Results indicated that the NHS-funded patients were more likely to score in the high risk SBT group than self-referred patients, but both groups had roughly the same percentage of patients scoring in the medium risk group. Self-referred patients were more likely to score in the low-risk group.

The Kongsted et al. [9] results suggested that both clinicians and the SBT have a relatively low ability to predict future pain outcomes [9]. Both accuratedly predicted a low percentage of patients who would be pain free (0/10 on a pain scale) at 2 weeks, but clinician predications were more likely than SBT to be associated with long-lasting LBP outcomes (83% of clinician predictions were associated with long-lasting outcomes versus 60% of SBT’s predictions). Clinician predictions were statistically associated with mean outcomes at all follow-up points, but not associated with accurate prediction of individuals’ outcomes. Combining the SBT with clinician predictions only slightly improved predictive accuracy.

The Morso et al. [21] study examined multiple variables across chiropractic, physiotherapy, and primary and secondary medical settings to determine which variables were related to the predictive utility of the SBT [21]. Current episode duration <2 weeks at care onset was the only variable that lowered the predictive ability of the SBT in all care settings. The chiropractic cohort had the greatest percentage of patients with short episode duration (chiropractic cohort = 62.1%, general practitioner cohort = 38.1%, physiotherapy cohort = 22.8%, secondary care cohort = 1.6%).

Across all reviewed studies, 26–59% of patients scored in the low risk category, 29–39% in the medium risk, and 10–39% in the high risk (Table 2). Relative to the studies conducted in primary care medical settings [8, 13], the study samples in the chiropractic settings reported greater percentages of patients whose episode duration was < 1 month.

## Discussion

The goal of this narrative review was to describe the findings of studies using the SBT in chiropractic settings. To date, no other reviews of this nature have been conducted. The findings suggest that it’s feasible to incorporate the SBT into chiropractic clinical settings [17] but that the short episode duration at onset of care (<2 weeks) with which many chiropractic patients present may be a barrier to the SBT’s predictive ability in some patients [9, 18, 21].

The Field and Newell [18] results indicate that high risk chiropractic patients may improve so quickly that there aren’t strong associations with SBT risk and poor outcomes. These results also suggest that there may be differences in patients with high risk in chiropractic settings compared to patients with high risk in primary care medical settings.

The Kongsted et al. [9] study revealed statistical association between the SBT score and long-lasting symptoms, but both clinicians’ and SBT predicted only 11 and 10% of patients, respectively, who would be pain free within 2 weeks of chiropractic care. This indicated poor predictive ability in both clinicians and SBT for short-term outcomes. The researchers in this study dichotomized the pain and disability variables, designating anything greater than 0 out of 10 on a pain scale or 8 out of 100 activity limitation as “poor outcomes.” This is quite a stringent standard, particularly in studying LBP; being completely pain free after 2 weeks of care isn’t a pragmatic goal and is beyond what is considered a clinically important improvement according to these outcome measures (the minimal clinically important difference ranges from 1.5 to 3.5 points of change for the NRS and 2.5 to 5 points of change for the RMDQ) [23–26]. Some patients in this study may have had clinically important improvements within 2 weeks (or later) but that data would have been lost in dichotomizing the outcome variables. Furthermore, though a greater percentage of clinicians compared to SBT accurately predicted which patients would have a long-lasting duration of symptoms, both predicted accurately more than 50% of the time for that variable. The SBT was weakest at predicting outcomes in participants who had a short duration of the LBP episode at onset of care, which describes a large percentage of the participants in this study.

The Morso et al. study results [21] suggest that short episode duration (<2 weeks) limits the SBT’s predictive ability across multiple care settings. The association between short duration and weak SBT prognostic accuracy may have played a role in the low prognostic utility in the Field and Newell [18] and Kongsted et al. [9] studies as well [9, 21, 18], although further studies are needed to better elucidate this phenomenon.

Since chiropractic settings showed the greatest percentage of short-duration episodes relative to other health care settings [21], chiropractors should be cautious when using the SBT on short episode duration patients. However, caution must also be exercised when interpreting mean group correlations, because the SBT may still be predictive in some short episode duration patients, as seen in the Newell et al. [20] study, despite not being associated with mean group outcomes.

Factors other than episode duration may also affect the SBT’s predictive ability. The Newell et al. [20] study demonstrated that the SBT was predictive in some patients who completed it 2 days after their initial treatment visit instead of at baseline. A study by Beneciuk et al. [27] also demonstrated that socioeconomic status, education level, and number of pain medications may affect the SBT’s predictive ability in medical care settings [28]. Research conducted in countries and subpopulations that differ from the 7 reviewed care settings will be important in better understanding the SBT’s predictive ability and utility for improving outcomes in patients seeking chiropractic care.

Other aspects of these studies also warrant attention. For example, a limitation of the Field and Newell [18] study is that participant data wasn’t collected beyond 3 months. Six and 12-month follow-up data would be valuable to see if a stronger correlation between SBT score and future prognosis existed at those time points. A limitation of the Field and Newell [18], Newell et al. [20], Kongsted et al. [9] and Morso et al. [21] studies is that the patients self-selected clinics in which they were treated. Self-selection may have ruled out many psychological barriers to improvement at the outset. Also, in the Field and Newell [18] study, roughly 41% of patients had been to the treating chiropractor in the past, which may have lead to greater expectation of improvement [18, 29, 30]. Future studies using the SBT might evaluate patients’ previous experience with manual therapy for their back pain and how expectations may affect outcomes.

Attending a course of manipulation may reduce psychological outcomes such as depression and mental component scores [19]. It’s possible that the inherent overlap between chiropractic treatment and the stratified care approach used in the STarT Back protocol altered outcomes in the studies examined in this review. Though the chiropractors in these studies were treating patients as usual and weren’t privy to patients’ SBT score, they may have altered or tailored their care based on clinical expertise and cues from patients about their psychological state, which is a stratification of sorts. This may have affected outcomes in ways that warrant further study.

All 7 reviewed studies were conducted in European populations (Denmark, Norway, and the UK) [9, 18–21, 22]. In the UK, it is unusual for chiropractic to be included in the state-funded health care system [27] and in Denmark only about 20% of costs are covered by the national health insurance [9]. Patients who seek chiropractic care in these countries may be in different socioeconomic strata than those in the general LBP population, or may have a different psychosocial risk profile from other countries [22, 27], which may affect the SBT’s predictive utility [28]. As demonstrated in the Field and Newell (2016) study [22], there also may be significant differences in patients who are self-referring for chiropractic care compared to those referred by general practitioners [27].

Furthermore, in the Irgens study [22], there was a statistically significant difference between the psychosocial risk profile in English versus Norwegian patients. Norwegian participants were younger, less distressed by their condition, and had lower catastrophization and depression, but were slightly more anxious than the UK participants studied. This lends further credence to the idea that the results of the reviewed studies may not be generalizable to patients in other countries where parallel analyses haven’t been conducted. Psychosocial risk profiles in other populations may differ enough to change the predictive ability of the SBT, or the outcome effects of stratified care pathways [22, 27].

However, none of the studies in this review implemented or examined STarT Back stratified care pathways. For patients who score in the low risk category, The STarT Back Protocol provides nothing more than a session of home advice, education about pain, and encouragement to continue physical activity [6]. Specific chiropractic treatment duration wasn’t described in these studies, except that treatment plans were unaffected by SBT categorization. It’s possible that some patients received minimal care, but it’s unknown. It’s not possible at this time to compare outcomes of usual chiropractic care to the SBT care pathway for low risk patients seeking chiropractic care. At present, the SBT remains a valuable tool for detecting low risk patients who may achieve comparable outcomes from one session of patient education and home exercises, instead of a course of spinal manipulation [5, 6, 12, 13].

Although the SBT was first developed to aid primary care medical physicians in determining best care pathways for patients with LBP, the SBT may be useful in stratifying care pathways for chiropractic clinicians. In many countries such as the US, Canada, and Great Britain, doctors of chiropractic are primary-contact providers [22, 31, 32]. Patients seeking care in chiropractic settings may benefit from stratified care in similar ways to those seeking medical care [5, 13] but, as demonstrated in this review, stratified care hasn’t yet been studied in chiropractic settings.

This review offers an assimilation of studies using the SBT in a chiropractic setting, but a number of limitations exist. First, this was a narrative review, so systematic methods of quality appraisal were not applied when selecting and reviewing manuscripts, and some articles may have been missed. One study was a secondary analysis, and no randomized controlled trials were available at the time of review. Therefore the strength of available evidence was low. Finally, letters to editors, conference proceedings, and other non-peer reviewed articles were excluded, which may have resulted in neglecting relevant information and discourse.

### Future research

Although the SBT lacked strong predictive value in short episode duration patients, perhaps a better focus for future research would be to explore the outcomes of stratified care in a broad range of chiropractic settings, particularly in light of the stratified care benefits reported in primary care medical settings [5, 13]. As the Morso et al. [21] study concluded, “the real potential of the SBT is as a tool for stratifying care pathways and therefore clinical trials are required to determine if the SBT is useful in chiropractic and secondary settings.”

## Conclusion

According to the current literature, the predictive ability of the SBT in medical and physical therapy patients translated only in some patients in some chiropractic settings, possibly due to the short episode duration with which many chiropractic patients present. In studies within medical and physiotherapy settings, stratification resulted in improved outcomes such as pain, disability, fear avoidance beliefs, and depression [5, 12, 13], but parallel studies in chiropractic settings have not been conducted. For that reason, regardless of the SBT’s questionable prognostic ability in the chiropractic patient populations studied, research examining the relationship between care stratification and outcomes in a chiropractic population would be valuable.

## Abbreviations

BQ: Bournemouth questionnaire
CSQ: Coping strategies questionnaire
FABQ: Fear avoidance beliefs questionnaire
LBP: Low back pain
MDI: Major depression inventory
NRS: Numerical rating scale
PGIC: Patient global impression of change
RMDQ: Roland morris disability questionnaire
SBT: STarT back tool
